# Magnetism in semiconducting molybdenum dichalcogenides

**DOI:** 10.1126/sciadv.aat3672

**Published:** 2018-12-21

**Authors:** Z. Guguchia, A. Kerelsky, D. Edelberg, S. Banerjee, F. von Rohr, D. Scullion, M. Augustin, M. Scully, D. A. Rhodes, Z. Shermadini, H. Luetkens, A. Shengelaya, C. Baines, E. Morenzoni, A. Amato, J. C. Hone, R. Khasanov, S. J. L. Billinge, E. Santos, A. N. Pasupathy, Y. J. Uemura

**Affiliations:** 1 Department of Physics, Columbia University, New York, NY 10027, USA.; 2 Laboratory for Muon Spin Spectroscopy, Paul Scherrer Institute, CH-5232 Villigen PSI, Switzerland.; 3 Department of Applied Physics and Applied Mathematics, Columbia University, New York, NY 10027, USA.; 4 Department of Chemistry, University of Zurich, Winterthurerstrasse 190, CH-8057 Zurich, Switzerland.; 5 School of Mathematics and Physics, Queen’s University Belfast, Belfast, UK.; 6 Department of Mechanical Engineering, Columbia University, New York, NY 10027, USA.; 7 Department of Physics, Tbilisi State University, Chavchavadze 3, GE-0128 Tbilisi, Georgia.; 8 Andronikashvili Institute of Physics of I. Javakhishvili Tbilisi State University, Tamarashvili str. 6, 0177 Tbilisi, Georgia.; 9 Condensed Matter Physics and Materials Science Department, Brookhaven National Laboratory, Upton, NY 11973, USA.

## Abstract

Transition metal dichalcogenides (TMDs) are interesting for understanding the fundamental physics of two-dimensional (2D) materials as well as for applications to many emerging technologies, including spin electronics. Here, we report the discovery of long-range magnetic order below *T*_M_ = 40 and 100 K in bulk semiconducting TMDs 2H-MoTe_2_ and 2H-MoSe_2_, respectively, by means of muon spin rotation (μSR), scanning tunneling microscopy (STM), and density functional theory (DFT) calculations. The μSR measurements show the presence of large and homogeneous internal magnetic fields at low temperatures in both compounds indicative of long-range magnetic order. DFT calculations show that this magnetism is promoted by the presence of defects in the crystal. The STM measurements show that the vast majority of defects in these materials are metal vacancies and chalcogen-metal antisites, which are randomly distributed in the lattice at the subpercent level. DFT indicates that the antisite defects are magnetic with a magnetic moment in the range of 0.9 to 2.8 μ_B_. Further, we find that the magnetic order stabilized in 2H-MoTe_2_ and 2H-MoSe_2_ is highly sensitive to hydrostatic pressure. These observations establish 2H-MoTe_2_ and 2H-MoSe_2_ as a new class of magnetic semiconductors and open a path to studying the interplay of 2D physics and magnetism in these interesting semiconductors.

## Teaser

Our experiments unambiguously establish 2H-MoTe_2_ and 2H-MoSe_2_ as magnetic, moderate bandgap semiconductors.

## INTRODUCTION

Transition metal dichalcogenides (TMDs), a family of two-dimensional (2D) layered materials like graphene, have been a subject of tremendous amounts of experimental and theoretical studies due to their exciting electronic and optoelectronic properties (*1*–*8*). TMDs share the same formula, *MX*_2_, where *M* is a transition metal and *X* is a chalcogen. They have a layered structure and crystallize in several polytypes, including 2H-, 1T-, 1T′-, and *T*_d_-type lattices (*9*). Much interest has focused on the cases of *M* = Mo or W, because the 2H forms of these compounds are semiconducting and can be mechanically exfoliated to a monolayer. In bulk form, 2H-MoTe_2_ is a semiconductor with an indirect bandgap of 0.88 eV. The unique properties of TMDs especially in the monolayer form have shown great promise in device applications such as magnetoresistance and spintronics, high on/off ratio transistors, optoelectronics, valley optoelectronics, superconductors, and hydrogen storage (*5*, *6*). Many of these interesting properties arise on account of the strong spin-orbit interaction present in these materials due to the heavy metal ion. While there are many studies focused on the spin-orbit coupling and the interesting consequences for electrical and optical properties in these systems, there are very limited, and mostly theoretical, studies on intrinsic magnetism in these materials (*10*–*18*). Theoretical and experimental work shows that in the absence of crystalline imperfections, the Mo-based TMDs are nonmagnetic (*10*). The ability to add magnetism into the properties of these materials can open up a host of new opportunities as tunable magnetic semiconductors.

In this paper, we report muon spin relaxation/rotation (μSR) and scanning tunneling microscopy (STM) experiments carried out on both polycrystalline and single-crystalline samples of 2H-MoTe_2_ and 2H-MoSe_2_, as well as Hubbard-corrected density functional theory calculations (DFT + *U*) to gain insights into the experimental results. μSR experiments serve as an extremely sensitive local probe technique to detect small internal magnetic fields and ordered magnetic volume fractions in magnetic materials. STM has the ability to measure atomic and electronic structure with atomic resolution, and has been used extensively in the past to study local electronic properties in TMDs and other 2D materials (*19*). The techniques of STM and μSR complement each other ideally, as we are able to study the magnetic properties of these crystals sensitively with μSR experiments and correlate these magnetic properties with atomic and electronic structure measured by STM. Experimental details are provided in Materials and Methods.

## RESULTS

### μSR experiments

**Fig. 1:**
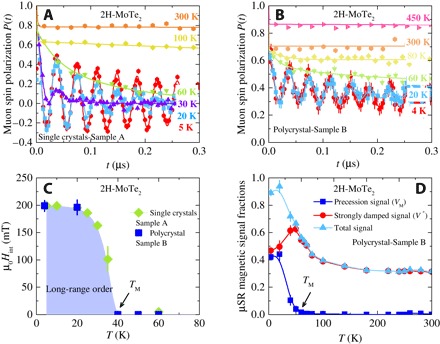
ZF μSR time spectra and temperature-dependent ZF μSR parameters for MoTe_2_. ZF μSR time spectra for the single-crystal (**A**) and polycrystalline (**B**) samples of MoTe_2_ recorded at various temperatures up to 450 K. (**C**) Temperature dependence of the internal field μ_0_*H*_int_ of 2H-MoTe_2_ as a function of temperature. (**D**) Temperature dependence of the magnetic fractions *V*_M_ and V* of the precession and strongly damped signals, respectively (see text). The total signal is also shown.

Zero-field (ZF) μSR time spectra for single-crystalline (sample A) and polycrystalline (sample B) samples of MoTe_2_, recorded for various temperatures in the range between 4 and 450 K, are shown in Fig. 1, A and B, respectively. At the highest temperature *T* = 450 K (Fig. 1B), nearly the whole sample is in the paramagnetic state. The paramagnetic state causes only a very weak depolarization of the μSR signal. This weak depolarization and its Gaussian functional form are typical for a paramagnetic material and reflect the occurrence of a small Gaussian Kubo-Toyabe depolarization, originating from the interaction of the muon spin with randomly oriented nuclear magnetic moments. Upon cooling, first, a fast decaying μSR signal is found. Below *T*_M_ ≃ 40 K, in addition to the strongly damped signal, a spontaneous muon spin precession with a well-defined frequency is observed, which is visible in the raw data (Fig. 1, A and B). Figure 1C shows the temperature dependence of the local magnetic field μ_0_*H*_int_ at the muon site for both single-crystalline and polycrystalline samples of MoTe_2_. There is a smooth increase of μ_0_*H*_int_ below *T*_M_ ≃ 40 K, reaching the saturated value of μ_0_*H*_int_ = 200 mT at low temperatures. Observation of the spontaneous muon spin precession indicates the occurrence of long-range static magnetic order in semiconducting 2H-MoTe_2_ below *T*_M_ ≃ 40 K, a remarkable finding. It is important to note that the long-range magnetic order was also observed in single-crystalline and polycrystalline samples of 2H-MoSe_2_ (see fig. S1), but with higher ordering temperature *T*_M_ ≃ 100 K and with the higher local magnetic field μ_0_*H*_int_ ≃ 300 mT. This difference might be related to the different magnetic structures in 2H-MoSe_2_ than in 2H-MoTe_2_. Figure 1D exhibits the temperature dependence of the μSR signal fractions (oscillating and strongly damped) in the polycrystalline sample of MoTe_2_. At the base *T* = 4 K, oscillations are observed for about 45% of the muons, while about 45% show a strong relaxation. In addition, it is evident from the ZF μSR data that the oscillating component develops at the cost of the strongly damped fraction, because the appearance of the oscillating component below *T* = 40 K is accompanied by the reduction of the strongly damped fraction (see Fig. 1D). The strongly damped signal observed in these compounds is most likely caused by the presence of some muonium fraction in semiconducting 2H-MoTe_2_. Muonium, a bound state of μ^+^ and an electron, may form in semiconductors (*20*). In the bound state, the muon is much more sensitive to magnetic fields than as a free probe, because its magnetic moment couples to the much larger electron magnetic moment, thus amplifying the depolarization effects. Therefore, even small variations in the magnetic field may cause a strong depolarization such as that observed in the spectra at early times. However, the presence of the oscillating signal in the ZF μSR time spectra in these compounds is a clear signature of magnetism with about 40% of magnetically ordered fraction.

**Fig. 2:**
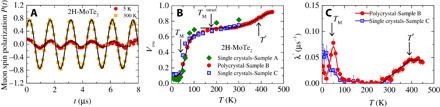
Temperature-dependent weak-TF μSR parameters and weak-TF μSR spectra for MoTe_2_. (**A**) WTF μSR time spectra for MoTe_2_ recorded at *T* = 5 and 300 K. The solid gray lines represent fits to the data by means of Eq. 2. Temperature dependence of the oscillating fraction (**B**) and the paramagnetic relaxation rate λ (**C**) of the single-crystalline and polycrystalline samples of MoTe_2_ obtained from the weak-TF μSR experiments. The solid arrows mark the magnetic transition temperatures *T*_M_ and T*. The solid gray lines represent fits to the data by means of phenomenological function (see Eq. 3 in Materials and Method).

To obtain precise information about the ordered magnetic volume fraction of MoTe_2_, weak–transverse field (TF) μSR experiments were carried out. In weak-TF experiments, the amplitude of the low-frequency oscillations of the muons precessing in the applied field is proportional to the non–magnetically ordered volume fraction. Thus, a spectrum with no oscillation corresponds to a fully ordered sample, while a spectrum with oscillation in the full asymmetry indicates a nonmagnetic sample. The weak-TF spectra for MoTe_2_, recorded at *T* = 4 and 300 K, are shown in Fig. 2A, which show intermediate oscillation amplitudes, indicating that, over a broad temperature interval, this material contains both magnetic and nonmagnetic regions. This compound therefore exhibits intrinsic nonmagnetic and magnetic phase separation. Figure 2B displays the fraction of the low-frequency oscillations *V*_osc_ = 1 − $A_{S}^{\prime }$(*T*)/*A*_S_(0) (see the analysis sections) as a function of temperature in two different single-crystalline (4 K ≤ *T* ≤ 450 K) and polycrystalline (4 K ≤ *T* ≤ 300 K) samples of MoTe_2_. At 450 K, *V*_osc_ exhibits nearly a maximum value, indicating that the whole sample is in the nonmagnetic state, and all the muon spins precess in the applied magnetic field. *V*_osc_ decreases with decreasing temperature below 425 K and tends to saturate below 300 K. This changes below 100 K, where an additional substantial decrease of *V*_osc_ is observed to value *V*_osc_ ≃ 0.1 below 40 K. The temperature dependence of the fraction *V*_osc_ is in fair agreement with the total μSR signal fraction, obtained from the ZF μSR experiments (Fig. 1D). The relaxation rate λ′ of the paramagnetic part of the signal (see the analysis sections for the details) also shows significant features in its temperature dependence. There is a clear peak at *T*_M_ ≃ 40 K, which is a signature of a magnetic phase transition. In the large temperature range beyond the peak, λ′ is constant and starts to increase above 250 K, reaching its maximum value at T* ≃ 400 K. This supports the presence of some transition in the sample at T* ≃ 400 K. It is important to emphasize that the weak-TF data obtained for two single-crystal samples agree very well with each other. Moreover, the data for the polycrystalline MoTe_2_ sample are also in excellent agreement with the single-crystal data. This implies that the observed transitions and long-range magnetic ordering below 40 K are reproducible and real. The combination of the ZF and TF μSR experiments allows us to conclude that there are two phases in the system 2H-MoTe_2_: (i) a low-*T* phase, which is characterized by the long-range static magnetic order (*T*_M_ ≃ 40 K), and (ii) a second phase, which appears below T* ≃ 400 K. This high-*T* transition can arise for few different reasons and it is most likely not magnetic in origin. This implies that only about 40% of the sample exhibits the magnetic order.

**Fig. 3:**
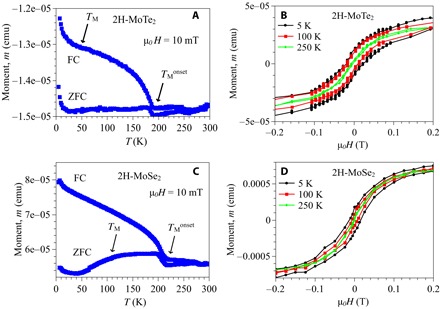
Temperature- and field-dependent magnetization data for MoTe_2_ and MoSe_2_. The temperature dependence of ZFC and FC magnetic moments of MoTe_2_ (**A**) and MoSe_2_ (**C**), recorded in an applied field of μ_0_*H* = 10 mT. The arrows mark the onset of the difference between ZFC and FC moment as well as the anomalies seen at low temperatures. The field dependence of magnetic moment of MoTe_2_ (**B**) and MoSe_2_ (**D**), recorded at various temperatures.

To substantiate the presence of magnetism in MoTe_2_ and MoSe_2_, we carried out the temperature- and field-dependent magnetization experiments. The temperature dependence of the macroscopic magnetic moment, recorded in an applied field of 10 mT under ZF-cooled (ZFC) (sample was cooled down to the base *T* in zero magnetic field, and the measurements were done upon warming) and field-cooled (FC) conditions (the sample was cooled down to the base *T* in an applied magnetic field, and the measurements were done upon warming) for MoTe_2_ and MoSe_2_, is shown in Fig. 3 (A and C) (the details are given in the Supplementary Materials). The field dependence of the magnetic moment for MoTe_2_ and MoSe_2_, recorded at three different temperatures, is shown in Fig. 3 (B and D). The difference between the ZFC and FC response and large hysteresis loop is observed for both samples at the base temperature, confirming the presence of ferromagnetism in these semiconductors. The magnitude of the loop decreases with increasing temperature and fully closes at high temperatures. The coercive field, estimated at *T* = 5 K, is 300 and 400 G for MoSe_2_ and MoTe_2_, respectively. The onset temperatures of the hysteresis (230 and 180 K for MoSe_2_ and MoTe_2_, respectively) are close to the temperature $T_{M}^{\text{onset}}$ below which μSR experiments show the appearance of inhomogeneous (short-range) magnetism (Fig. 2B and fig. S2). This means that below $T_{M}^{\text{onset}}$, some small ferromagnetic domains form, which produces inhomogeneous magnetic fields in the samples. μSR experiments reveal a well-defined uniform magnetism below *T*_M_ ≃ 40 and 100 K for MoTe_2_ and MoSe_2_, respectively, and the anomalies (such as an additional increase of the moment and of the difference) at around these temperatures can also be seen in magnetization data (Fig. 3, A and C). The SQUID data are consistent with the μSR results and can be considered as additional independent piece of evidence for the presence of magnetism in MoTe_2_ and MoSe_2_.

### STM experiments

**Fig. 4:**
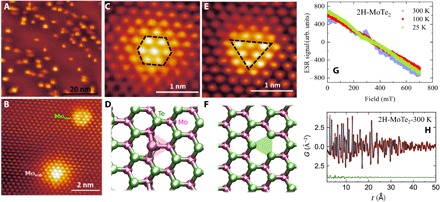
Observation of intrinsic defects in 2H-MoTe_2_ through STM and sample characterizations. (**A**) Large-scale atomic-resolution STM topography (20 nm) of the MoTe_2_ surface. The image reveals an approximately uniform density of two types of defects over the entire surface. The STM topography was taken at −1.25 V and −100 pA set point. (**B**) Small-scale atomic-resolution STM topography (2 nm) shows that these two types of defects are mainly substitutional Mo atoms at Te sites (Mo_sub_) and Mo vacancies (Mo_vac_). (**C** and **D**) Local STM topography (1 nm) and DFT + *U*–optimized geometry for Mo_sub_ defect, respectively. The observed atoms in (C) are those at the top layer of tellurium, with an increased topographic height profile at the center of the six brightest spots. We attribute this to a molybdenum replacement of a tellurium atom. (**E** and **F**) Local-scale STM topography (1 nm) and DFT + *U*–optimized geometry of the second type of defects observed, respectively. The image in (E) shows a depression in the topographic height profile, centered between three tellurium atoms. On the basis of the symmetry, we attribute this to a molybdenum vacancy under the layer of tellurium. (**G**) ESR spectra for 2H-MoTe_2_, recorded at various temperatures. (**H**) PDF average structure refinements for 2H-MoTe_2_ at 300 K fitted to the hexagonal 2H-structure model.

Previous theoretical work (*10*) and simple chemical bonding considerations indicate that the Mo atoms in 2H-MoTe_2_ are in a nonmagnetic *4d^2^* configuration. We therefore investigate the presence of defects in the crystals measured by μSR and ask whether we can associate them with the observed magnetism. To probe this possibility, we perform atomic-resolution STM measurements on in situ cleaved surfaces on crystals from the same batch from which μSR measurements were performed. Figure 4A shows a typical STM topograph of the surface of MoTe_2_. The density of defects observed is low enough that they can simply be counted individually to make an estimate of the defect density in the crystal. The defect density measured in Fig. 4A is 0.4%, a number that is typical of all samples measured. To understand these defects further, high-resolution STM imaging was performed to resolve the lattice site at which defects are observed. Being a local surface probe, STM imaging resolves only the top tellurium of the MoTe_2_ lattice clearly. We use the Te atoms to infer defect locations based on their relative heights and centers. Two types of defects were observed within these crystals: one located on the Te site (Fig. 4, B to D) and the other located at the metal site (Fig. 4, B, E, and F). From the relative densities of these two defects, we find a majority of sites to be the former Te site defect. We see from the topography of this defect that the defect is associated with a foreign atom replacing the chalcogen rather than a chalcogen vacancy. While STM measurements themselves cannot identify the chemical nature of the defect, we have performed electron spin resonance (ESR) experiments to investigate the presence of foreign ferromagnetic impurities such as Fe or Ni. The results of these measurements are shown in Fig. 4G (see also fig. S2), which indicate no trace of ESR signal down to the lowest temperature. Previous transmission electron microscopy (TEM) measurements (*19*) have also shown that one of the prominent defect types in these materials is a chalcogen antisite, where a molybdenum atom replaces the tellurium atom. We therefore proceed to identify it as such. The other type of defect (Fig. 4, E and F), which is only present in low density, is at the site of the metal atom. Based also on a combination of STM and TEM measurements (*21*) of defects, we identify this with a Mo vacancy (Mo_vac_) in the crystal. The TEM imaging provides chemical identification that shows that the most prominent defect on the metal site is the vacancy. The STM measurements are more accurate than TEM at determining the counts of the vacancy defects, and the obtained counts are consistent between STM and TEM. This identification is also consistent with STM images, which show that the vacancy is a topographic depression at all biases measured. Note that two major defects such as metal vacancies and chalcogen antisites were found (*21*) in these materials synthesized by two different methods—chemical vapor transport and self-flux growth. Commercial samples were also tested and the same types of defects were seen, indicating that defects are really intrinsic for MoTe_2_ and MoSe_2_. Formation energies for a metal vacancy and for the metal antisite defects were found (*21*) to be 5.22 and 4.81 eV, respectively. Although chalcogen vacancies have lower formation energies, they are almost never observed in our crystals, likely due to the fact that, during growth, very large ratios of chalcogen to metal sources are used (usually on the order of 100:1) in both commercially grown and homegrown crystals. Similar results were obtained for MoTe_2_ at similar defects: Metal antisite gives a formation energy of 4.68 eV, while metal vacancies give a formation energy of 10.62 eV, which is appreciably larger than MoSe_2_. This suggests that most of the magnetic defects may be antisite in the case of MoTe_2_ samples. Last, average structure pair distribution function (PDF) refinement for 2H-MoTe_2_ (Fig. 4H) (see also fig. S3) confirms the 2H polytype (SG:*P*6_3_/*mmc*) (*9*) and shows no evidence of structural distortions or segregation, consistent with the dilute concentration of intrinsic defects. This is in line with the observation of a random distribution of defects from STM.

### DFT calculations

**Fig. 5:**
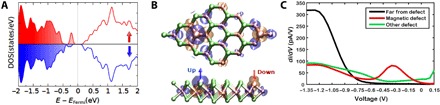
DFT + *U* and STM. (**A**) Spin-polarized density of states, DOS(states/eV), of Mo_sub_ defects in the antiferromagnetic (AFM) phase. Fermi level, *E*_Fermi_, is set to zero. Both the spin-up and spin-down DOS reveal an in-gap state due to the defect. (**B**) Magnetization density (±0.001 electrons/Bohr^3^) on the top surface of bulk 2H-MoTe_2_ in AFM configuration. Spin-up and spin-down states are shown in faint blue and orange isosurfaces, respectively. Note that spins also couple antiferromagnetically at the local level between the Mo impurity and the nearest Mo atoms. (**C**) Scanning tunneling spectroscopy *dI*/*dV*s taken on the two types of defect as well as far from any defect.

Having identified the primary defect types in our crystals, we perform DFT + *U* (see Materials and Methods for details) to examine their magnetic properties. In the absence of the Hubbard *U*, the defects are found to be nonmagnetic. At finite values of *U*, a magnetic moment in the range of 0.9 to 2.8 μ_B_ is observed per Mo antisite impurity. Along with magnetism, the calculations find that, in the presence of *U*, there are small distortions from triangular symmetry at the Mo_sub_ defect site. The spin-polarized density of states (Fig. 5A) shows that the localized Mo 4*d* states at the Fermi level carry most of the magnetization with minor contribution from *p* states of the Te atoms. We also find that the Mo_sub_ defects are coupled antiferromagnetically to the nearest-neighbor Mo atoms, as shown in Fig. 5B. The magnetic moments at the nearest-neighbor Mo atoms can reach 0.10 to 0.40 μ_B_/atom, with smaller contributions for second and third neighbors (0.02 to 0.08 μ_B_/atom). The Te atoms show negligible spin polarization. Similar effects have previously been observed in graphene with different adsorbates and substitutional metal atoms (*22*, *23*). The metal vacancy Mo_vac_ does not introduce a significant local moment in our calculations. Figure 5C shows STM tunneling spectra taken on the two types of defect as well as far from any defect. Spectra taken on the Mo_sub_ defects always display a deep in-gap state, while the metal vacancy Mo_vac_ does not show such a feature. Compared to the DFT calculation in Fig. 5A, we see that this in-gap state is consistent with the DFT model for a molybdenum replacement of a tellurium.

### Pressure-dependent magnetism

**Fig. 6:**
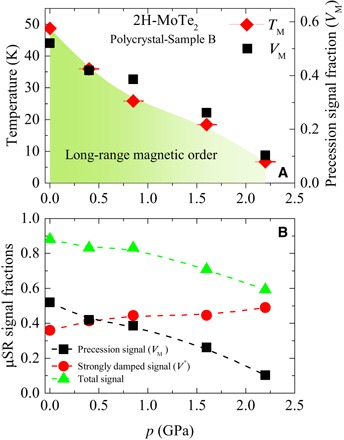
Pressure evolution of various quantities. (**A**) Magnetic transition temperature *T*_M_ and magnetic volume fraction *V*_M_ as a function of pressure. (**B**) Pressure dependence of the magnetic fractions *V*_M_ and V*, corresponding to the precession μSR signal and the strongly damped μSR signal, respectively. The total magnetic signal is also shown. The dashed lines are guides to the eyes.

For further insight into the magnetic order in MoTe_2_, weak-TF μSR experiments were carried out as a function of hydrostatic pressure. Namely, the temperature dependence of the paramagnetic fraction was measured for MoTe_2_ at various applied pressures up to 2.2 GPa (see fig. S5). On the basis of these data, we can construct a temperature-pressure phase diagram for 2H-MoTe_2_. Figure 6 (A and B) shows the pressure dependences of the magnetic transition temperature *T*_M_ and the magnetic volume fractions *V*_M_ and V* (*T* = 100 K). We find that hydrostatic pressure has a significant effect on the magnetic properties of these materials. Namely, we see a suppression of the magnetic order as a function of pressure, which can be observed as the amplitude of the low-temperature transition decreases with pressure (fig. S5), while the strongly damped signal (fraction, estimated at temperatures above the onset of long-range magnetic transition from fig. S5) remains relatively uniform, as shown in Fig. 6B. Further, it is clear from Fig. 6A that the transition temperature *T*_M_ (midpoint of the transition, indicated by the arrows in fig. S5) decreases as a function of pressure. These findings show that one can physically tune the magnetism in these materials with pressure. Strong pressure dependence of magnetism is very encouraging, because it implies that one can have control over the magnetic properties. This also means that uniaxial strain, which is another interesting and widely applied tuning parameter, might also be useful in these materials. Currently, we do not yet adequately understand this behavior to comment further, but this strong pressure dependence may be related to the following effects: (i) pressure-induced metallicity in 2H-MoTe_2_, as shown previously from resistivity experiments (*4*), and (ii) enhanced interlayer coupling, caused by the reduction of *c* axis with pressure. Further experimental and theoretical efforts are certainly needed for understanding pressure-dependent data.

## DISCUSSION

Our muon measurements unambiguously establish 2H-MoTe_2_ and 2H-MoSe_2_ as magnetic, moderate bandgap semiconductors. The μSR results indicate magnetic order below *T*_M_ ≃ 40 and 100 K for MoTe_2_ and MoSe_2_, respectively. In the same materials, STM measurements show the presence of intrinsic dilute self-organized magnetic tellurium/selenium antisite defects, a finding that is well supported by Coulomb-corrected DFT. Although the exact link between μSR and STM/DFT results is not yet clear, both results together constitute first strong evidence for the involvement of magnetic order in physics of TMDs. Establishing long-range magnetic order with the observed low density of antisite defects would necessarily involve electronic coupling to the semiconductor valence electrons. The presence of such spin-polarized itinerant electrons implies that these materials are dilute magnetic semiconductors (DMSs). Previously, DMS materials have been synthesized in a range of thin film (*24*) and crystal (*25*) materials. Much interest has centered on the III-V semiconductor class (*24*, *26*–*28*), where a small concentration of some magnetic ions, particularly Mn^2+^, can be incorporated by substituting for the group III cations of the host semiconductor. Numerous technical challenges in making uniform DMS materials have been overcome in recent years (*25*, *29*–*31*), but formidable challenges still remain in producing stable, high-quality DMS materials with high *T*_c_. Our present system offers unique advantages to these other routes to synthesize DMS materials. First, the defects contributing to magnetism are intrinsic in the crystal and are uniformly distributed. This can alleviate some of the materials challenges commonly faced in DMS synthesis. Second, the materials are cleavable and readily grown in large-area form down to a monolayer thickness (*32*). As is well known in these materials, the bandgap is a strong function of thickness, giving us tunability over the semiconductor properties. Third, the chemical potential and electric field in thin films are easily tuned by electrostatic gates (*33*), allowing the possibility of tunable magnetism as has been seen for GaAs (*24*, *27*). Last, these materials can be easily layered by van der Waals heteroepitaxy (*34*), allowing the creation of unique new device concepts in the future. Recently, there was a very interesting finding of ferromagnetism in VSe_2_ monolayers. However, that monolayer and few-layers VSe_2_ are metals with high density of states at the Fermi level (*8*). The novelty of our work is the fact that we see intrinsic long-range magnetic order at the same time as good semiconducting behavior in 2H-MoTe_2_ and 2H-MoSe_2_. At present, we do not have a systematic understanding of how the defect concentration in these materials determines the magnetic transition temperature and saturation magnetization. We also have limited control over the number of defects in these samples. To fully exploit the magnetic properties of these TMD semiconductors, future work needs to address these important issues.

## MATERIALS AND METHODS

### Sample preparation

High-quality single crystals and polycrystalline samples were obtained by mixing molybdenum foil (99.95%) and tellurium lumps (99.999+%) in a ratio of 1:20 in a quartz tube and sealed under vacuum. The reagents were heated to 1000°C within 10 hours. They dwelled at this temperature for 24 hours, before they were cooled to 800°C within 30 hours (polycrystalline sample) or 100 hours (single crystals). At 800°C, the tellurium flux was spinned off and the samples were quenched in air. The obtained MoTe_2_ samples were annealed at 400°C for 12 hours to remove any residual tellurium.

### Pressure cell

Pressures of up to 2.2 GPa were generated in a double-wall piston-cylinder type of cell made of CuBe and MP35N materials, especially designed to perform μSR experiments under pressure (*35*, *36*). As a pressure-transmitting medium, Daphne oil was used. The pressure was measured by tracking the SC transition of a very small indium plate by AC susceptibility. The filling factor of the pressure cell was maximized. The fraction of the muons stopping in the sample was approximately 40%.

### μSR experiment

In a μSR experiment, nearly 100% spin-polarized muons μ^+^ are implanted into the sample one at a time. The positively charged μ^+^ thermalize at interstitial lattice sites, where they act as magnetic microprobes. In a magnetic material, the muon spin precesses in the local field μ_0_*H*_int_ at the muon site with the Larmor frequency ν_μ_ = μ_0_γ_μ_/(2π)*H*_int_ [muon gyromagnetic ratio γ_μ_/(2π) = 135.5 MHz T^−1^].

μSR experiments under pressure were performed at the μE1 beamline of the Paul Scherrer Institute (Villigen, Switzerland), where an intense high-energy (*p*_μ_ = 100 MeV/c) beam of muons is implanted in the sample through the pressure cell. The low-background GPS (πM3 beamline) (*37*) and low-temperature LTF (πM3.3) instruments were used to study the single-crystalline and polycrystalline samples of MoTe_2_ at ambient pressure.

### Analysis of the ZF-μSR data of MoTe_2_

Within the whole temperature range, the response of the sample consists of a magnetic and nonmagnetic contribution. At low temperatures, description of the magnetic part of the μSR signal requires a two-component relaxation function. For temperatures below 50 K, a well-defined frequency is observed for about 45% of the muons, while about 45% show a strong relaxation due to a broad static field distribution. This situation changes above 50 K, where only the strongly damped signal is observed in addition to the paramagnetic signal. The data were analyzed by the following functional form using the free software package MUSRFIT (*38*)

$$\begin{array}{c} P_{S}(t)=V_{M}[\frac{2}{3}e^{-\lambda_{T}t}\text{cos}(\gamma_{\mu }\mu_{0}H_{\text{int}}t)+\frac{1}{3}e^{-\lambda_{L}t}]+\\ V*\frac{2}{3}e^{-\lambda_{\text{Fast}}t}+(1-V_{M}-V*)e^{-\lambda_{\text{nm}}t} \end{array}$$ (1)

Here, *V*_M_ and V* denote the relative magnetic fraction of the oscillating and strongly damped magnetic signals, respectively. μ_0_*H*_int_ is the local internal magnetic field at the muon site. λ_T_ and λ_L_ are the depolarization rates representing the transversal and longitudinal relaxing components of the magnetic parts of the sample. λ_nm_ is the relaxation rate of the nonmagnetic part of the sample.

### Analysis of the weak TF-μSR data of MoTe_2_

A substantial fraction of the μSR asymmetry originates from muons stopping in the pressure cell surrounding the sample (*35*). Therefore, the μSR data in the whole temperature range were analyzed by decomposing the signal into a contribution of the sample and a contribution of the pressure cell. In addition, the TF-μSR spectra were fitted in the time domain with a combination of a slowly relaxing signal with a precession frequency corresponding to the applied field of μ_0_*H* = 5 mT (due to muons in a paramagnetic environment) and a fast relaxing signal due to muons precessing in much larger static local fields:

$$\begin{array}{c} A_{0}P(t)=A_{S}(0)P_{S}(t)+A_{\text{PC}}(0)P_{\text{PC}}(t)=\\ (A_{\text{PC}}e^{-\lambda_{\text{PC}}t}+A_{S}^{\prime }e^{-\lambda \prime t})\text{cos}(\gamma_{\mu }B\prime t)+\\ A_{S}^{\prime \prime }[\frac{2}{3}e^{-\lambda_{T}^{\prime \prime }t}J_{0}(\gamma_{\mu }B^{\prime \prime }t)+\frac{1}{3}e^{-\lambda_{L}^{\prime \prime }t}] \end{array}$$ (2)

where *A*_0_ is the initial asymmetry, that is, the amplitude of the oscillation in the fully paramagnetic state. *P*(*t*) is the muon spin-polarization function, and γ_μ_/(2π) ≃ 135.5 MHz/T is the muon gyromagnetic ratio. *A*_PC_ and λ_PC_ are the asymmetry and the relaxation rate of the pressure cell signal. $A_{S}^{\prime }$ and $A_{S}^{\prime \prime }$ are the amplitudes of the slow (paramagnetic) and fast relaxing sample signals, respectively. λ′ is the relaxation rate of the paramagnetic part of the sample, caused by the paramagnetic spin fluctuations and/or nuclear dipolar moments. $\lambda_{T}^{\prime \prime }$ and $\lambda_{L}^{\prime \prime }$ are the transverse and longitudinal relaxation rates, respectively, of the magnetic part of the sample. *B*′ and *B*″ are the magnetic fields, probed by the muons stopped in the paramagnetic and magnetic parts of the sample, respectively. From these refinements, the paramagnetic fraction at each temperature *T* was estimated as *V*_osc_ = 1 − $A_{S}^{\prime }$(*T*)/*A*_S_(0).

### Analysis of the temperature dependence of *V*_osc_ in MoTe_2_

The values of *T*_M_ and T* were determined by using the phenomenological function (*39*)

$$A(T)/A_{0}=a[1-\frac{1}{\text{exp}[(T-T_{X})/\Delta T_{X}]+1}]+b$$ (3)

where *X* = *M*, M*. Δ*T*_*X*_ is the width of the transition, and *a* and *b* are empirical parameters. Analyzing the data in Fig. 2 with Eq. 3 yields *T*_M_ = 50(3) K and T* = 340(3) K.

### Ab initio DFT methods

Calculations were based on ab initio DFT using the VASP code (*40*). The generalized gradient approximation (*41*), together with van der Waals corrected functionals (*42*, *43*), was used. The latter guarantees if any modification of the dispersion coefficients due to the spin coupling would affect the interlayer distance. No appreciable variations were observed. A well-converged plane-wave cutoff of 800 eV was used in all calculations. Projected augmented wave (*44*, *45*) potentials have been used in the description of the bonding environment for Mo, Se, and Te. Atoms and cell volumes were allowed to relax until the residual forces were below 0.0001 eV/Å under the conjugate gradient algorithm. To model the system studied in the experiments, we created large supercells containing up to 300 atoms to simulate bulk 2H-MoTe_2_ and 2H-MoSe_2_ with different defects and concentrations. The Brillouin zone was sampled with a 3 × 3 × 2 grid under the Monkhorst-Pack scheme (*46*) to perform relaxations. Energetics and electronic density of states were calculated using a converged 11 × 11 × 3 *k*-sampling for the unit cell of 2H-MoTe_2_ and 2H-MoSe_2_. Calculations included a Hubbard-*U* correction (*47*) within the range of 0.5 to 4.0 eV to account for the strong on-site interactions on the doped system, in particular those due to the localized *d* states at the Fermi level. In addition to this, we used a Fermi-Dirac distribution with an electronic temperature of *k*_B_*T* = 20 meV to resolve the electronic structure.

## Supplementary Material

http://advances.sciencemag.org/cgi/content/full/4/12/eaat3672/DC1

## SUPPLEMENTARY MATERIALS

Supplementary material for this article is available at http://advances.sciencemag.org/cgi/content/full/4/12/eaat3672/DC1 Fig. S1. ZF μSR time spectra and temperature-dependent parameters for MoSe_2_. Fig. S2. The temperature dependence of the paramagnetic fraction for 2H-MoTe_2_ and 2H-MoSe_2_. Fig. S3. ESR signals for 2H-MoTe_2_ and 2H-MoSe_2_. Fig. S4. PDF results for 2H-MoTe_2_ and 2H-MoSe_2_. Fig. S5. Temperature and pressure evolution of the paramagnetic fraction *V*_osc_. Fig. S6. Magnetization data for MoSe_2_ and MoTe_2_. Fig. S7. Hysteresis loop for MoSe_2_ and MoTe_2_. Fig. S8. Calculated magnetization of the antisite defect versus Hubbard *U*. References (*48*–*53*)
