# Supplementary material for: Magnetism in semiconducting molybdenum dichalcogenides
Source: Sci Adv. 2018 Dec 21;4(12):eaat3672. doi: 10.1126/sciadv.aat3672 (PMC6303124; doi:10.1126/sciadv.aat3672)
Supplement: http://advances.sciencemag.org/cgi/content/full/4/12/eaat3672/DC1 [file supp_4_12_eaat3672__index.html]

Science Advances | Science Advances

## Supplementary Materials

**This PDF file includes:**

- Fig. S1. ZF μSR time spectra and temperature-dependent parameters for MoSe2.
- Fig. S2. The temperature dependence of the paramagnetic fraction for 2H-MoTe2 and 2H-MoSe2.
- Fig. S3. ESR signals for 2H-MoTe2 and 2H-MoSe2.
- Fig. S4. PDF results for 2H-MoTe2 and 2H-MoSe2.
- Fig. S5. Temperature and pressure evolution of the paramagnetic fraction *V*osc.
- Fig. S6. Magnetization data for MoSe2 and MoTe2.
- Fig. S7. Hysteresis loop for MoSe2 and MoTe2.
- Fig. S8. Calculated magnetization of the antisite defect versus Hubbard *U*.
- References (*48*–*53*)

Download PDF

**Files in this Data Supplement:**

- Adobe PDF - aat3672\_SM.pdf
