# Supplementary material for: Magnetism in semiconducting molybdenum dichalcogenides
Source: Sci Adv. 2018 Dec 21;4(12):eaat3672. doi: 10.1126/sciadv.aat3672 (PMC6303124; doi:10.1126/sciadv.aat3672)
Supplement: http://advances.sciencemag.org/cgi/content/full/4/12/eaat3672/DC1 [file aat3672_SM.pdf]

Science Advances
AAAS

advances.sciencemag.org/cgi/content/full/4/12/eaat3672/DC1

Supplementary Materials for

Magnetism in semiconducting molybdenum dichalcogenides

Z. Guguchia*, A. Kerelsky, D. Edelberg, S. Banerjee, F. von Rohr, D. Scullion, M. Augustin, M. Scully, D. A. Rhodes, Z. Shermadini, H. Luetkens, A. Shengelaya, C. Baines, E. Morenzoni, A. Amato, J. C. Hone, R. Khasanov, S. J. L. Billinge, E. Santos*, A. N. Pasupathy*, Y. J. Uemura*

*Corresponding author. Email: zurab.guguchia@psi.ch (Z.G.); e.santos@qub.ac.uk (E.S.); apn2108@columbia.edu (A.N.P.); tomo@lorentz.phys.columbia.edu (Y.J.U.)

Published 21 December 2018, *Sci. Adv.* **4**, eaat3672 (2018)
DOI: 10.1126/sciadv.aat3672

This PDF file includes:

Fig. S1. ZF μSR time spectra and temperature-dependent parameters for $MoSe_2$.
Fig. S2. The temperature dependence of the paramagnetic fraction for 2H-$MoTe_2$ and 2H-$MoSe_2$.
Fig. S3. ESR signals for 2H-$MoTe_2$ and 2H-$MoSe_2$.
Fig. S4. PDF results for 2H-$MoTe_2$ and 2H-$MoSe_2$.
Fig. S5. Temperature and pressure evolution of the paramagnetic fraction V_{osc}.
Fig. S6. Magnetization data for $MoSe_2$ and $MoTe_2$.
Fig. S7. Hysteresis loop for $MoSe_2$ and $MoTe_2$.
Fig. S8. Calculated magnetization of the antisite defect versus Hubbard U.
References (*48–53*)

SUPPLEMENTARY INFORMATION

Zero-field μSR data for $MoSe_2$

Zero-field μSR time spectra for the polycrystalline (Sample D) sample of $MoSe_2$, recorded for various temperatures in the range between 10 K and 300 K, are shown in Fig. S1. Below $T_M \simeq 100$ K, in addition to the strongly relaxed signal, a spontaneous muon-spin precession with a well-defined frequency is observed, which is clearly visible in the raw data. The inset of Fig. S1 shows the temperature dependence of the local magnetic field $\mu_0 H_{int}$ at the muon site for both single crystalline (Sample E) and polycrystalline (Sample D) samples of $MoSe_2$. There is a smooth increase of $\mu_0 H_{int}$ below T_M $\simeq 100$ K, reaching the saturated value of $\mu_0 H_{int} = 310$ mT at low temperatures. Observation of the spontaneous muon-spin precession indicates the occurrence of long range static magnetic order in semiconducting 2H-$MoSe_2$, similar as for 2H-$MoTe_2$. However, the magnetic ordering temperature $T_M \simeq 100$ K as well as the internal field $\mu_0 H_{int} \simeq 310$ mT in 2H-$MoSe_2$ is higher as compared to the ones $T_M \simeq 40$ K and $\mu_0 H_{int} \simeq 200$ mT, observed in 2H-$MoTe_2$. This difference might be related to the different magnetic structures in these two samples 2H-$MoSe_2$ than in 2H-$MoTe_2$. On the other hand, the fraction of the strongly damped signal is higher in 2H-$MoSe_2$ than in 2H-$MoTe_2$.

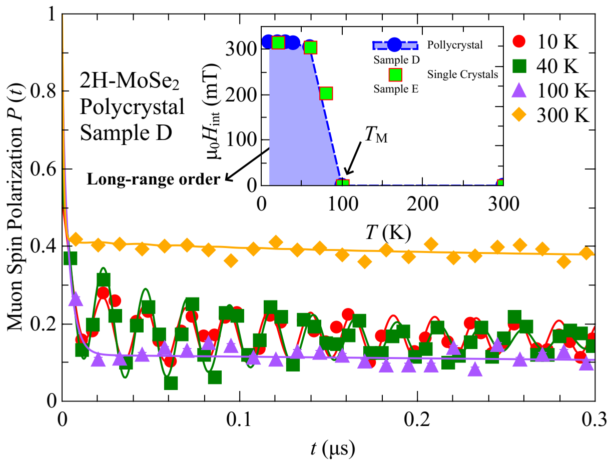

Fig. S1. (Color online) **ZF μSR time spectra and temperature-dependent parameters for $MoSe_2$.** ZF μSR time spectra for the polycrystalline samples of $MoSe_2$ recorded at various temperatures up to $T = 300$ K. The inset shows the internal field $H_{\rm int}$ of $MoSe_2$ as a function of temperature.

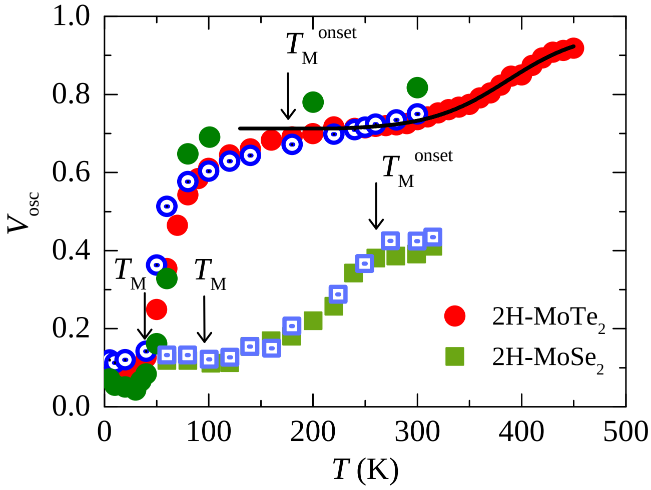

Fig. S2. (Color online) **Weak TF μSR results for $MoSe_2$ and $MoTe_2$. The temperature dependence of the paramagnetic fraction for 2H-$MoTe_2$ and 2H-$MoSe_2$.** Arrows mark the onset and long-range ordered temperatures for magnetism.

Zero-field μSR measurements reveal that the homogeneous internal magnetic fields in 2H-$MoTe_2$ and 2H-$MoSe_2$ appear below $T_M \simeq 40$ K and 100 K, respectively. However, the weak-TF μSR experiments show that the short-range (inhomogeneous) magnetism appear at much higher temperatures. Figure S2 displays the fraction of the low-frequency oscillations $V_{\rm osc}$, determined from the weak-TF μSR data, as a function of temperature in 2H-$MoTe_2$ and 2H-$MoSe_2$. In $MoTe_2$, at 450 K, $V_{\rm osc}$ exhibits nearly a maximum value and decreases with decreasing temperature and tends to saturate below 300 K. This $\sim$ 30 % reduction of $V_{\rm osc}$ can arise for few different reasons and it is most likely not magnetic in origin. However, there is an additional decrease of $V_{\rm osc}$ starting below $T_{\rm M}^{onset} \sim 180$ K, which is due to the appearance of the inhomogeneous short-range magnetism and $V_{\rm osc}$ continues to reduce until it reaches the minimum value $V_{\rm osc} \simeq$ 0.1 at the long-range magnetic ordering temperature T_M $\simeq 40$ K, below which zero-field μSR shows a well defined uniform internal magnetic fields. This implies that, the long range magnetic order is achieved only below $T_M \simeq$ 40 K, while the short range magnetism appear at higher temperature $T_{\rm M}^{onset} \sim 180$ K. The same is also observed in $MoSe_2$ (see Fig. S2), i.e., the short range magnetism appear below $T_{\rm M}^{onset} \sim 250$ K, while the long-range order is achieved below $T_M \simeq 100$ K. Such a big difference between the short range and long range magnetic ordering temperatures was also observed in antiferromagnetic $EuTiO_3$ [48].

Electron Spin Resonance measurements for 2H-$MoTe_2$ and 2H-$MoSe_2$

The polycrystalline samples of 2H-$MoTe_2$ and 2H-$MoSe_2$ have been studied by means of the Electron Spin Resonance (ESR) technique with the emphasis on checking for the paramagnetic impurity phases. ESR experiments were performed with a Bruker EMX spectrometer at X-band frequencies (ν = 9.4 GHz) equipped with a continuous He gas-flow cryostat in the temperature range $10 < T < 300$ K. In Figure S3a and b the ESR spectra of 2H-$MoTe_2$ and 2H-$MoSe_2$, respectively, are shown for various temperatures. It is remarkable that no trace of ESR signal was found in the sample 2H-$MoTe_2$ (see Fig. S3a) down to the lowest temperature, indicating the absence of any even small amount of paramagnetic impurities such as Fe or Ni. This provides strong support to the intrinsic nature of magnetic order in 2H-$MoTe_2$, observed by μSR. We

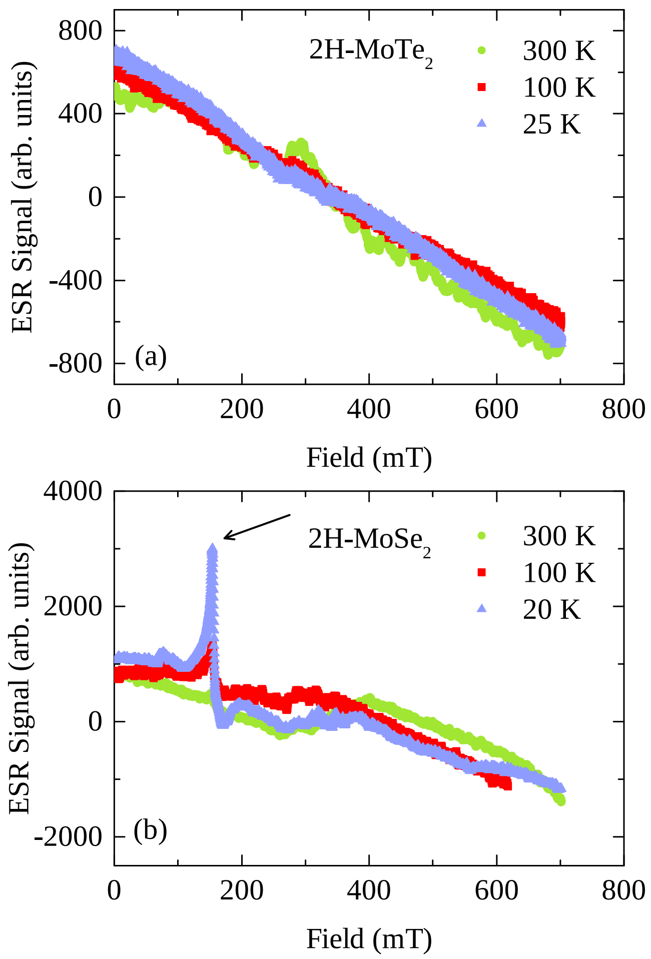

Fig. S3. (Color online) **ESR signals for 2H-$MoTe_2$ and 2H-$MoSe_2$.** Electron Spin Resonance spectra of polycrystalline samples of 2H-$MoTe_2$ (a) and 2H-$MoSe_2$ (b), taken at different temperatures. The arrow marks the ESR signal with a peak value corresponding to a g-factor of 4.

found a small ESR signal in 2H-$MoSe_2$ with the g-factor of 4 (See Fig. S3b), which indicates the presence of small amount of paramagnetic Fe-impurities. However, this tiny amount of paramagnetic Fe-impurities would not lead to the observed long-range magnetic order in 2H-$MoSe_2$. ESR experiments give evidence that the magnetic order in 2H-$MoTe_2$ and 2H-$MoSe_2$ is not related to the presence of an impurity phase but it is an intrinsic state.

Pair Distribution Function (PDF) Structure Confirmation of 2H-$MoTe_2$ and 2H-$MoSe_2$

Total scattering X-ray measurements were performed at the National Synchrotron Light Source II (XPD, 28-ID-2), Brookhaven National Laboratory. Finely ground powders of 2H-$MoTe_2$ and 2H-$MoSe_2$ were sealed in polyimide capillaries and diffraction patterns were collected in a Debye-Scherrer geometry with an X-ray energy of 66.479 keV (λ = 0.1865 Å) using a large-area 2D Perkin Elmer detector. The detector was mounted with a sample-to-detector distance of 204.14 mm. The samples were measured at 90 K and 300 K using an Oxford CS-700 cryostream. The experimental geometry, 2θ range, and detector misorientations were calibrated by measuring a crystalline nickel powder directly prior to data collection at each temperature point, with the experimental geometry parameters refined using the PyFAI program [49]. Standardized corrections are then made to the data to obtain the total scattering structure function, $F(Q)$, which is then Fourier transformed to obtain the PDF, using PDFgetX3 [50] within the xPDFsuite [51]. The maximum range of data used in the Fourier transform (Q_{max}, where $Q = 4\pi \sin\theta/\lambda$ is the magnitude of the momentum transfer on scattering) was chosen to be 25 $Å^{-1}$ to give the best tradeoff between statistical noise and real-space resolution. PDFGUI was used to construct unit cells from reference structures, carry out structure refinements, and determine the agreement between calculated PDFs and data, quantified by the residual, R_w [52].

Average structure PDF refinements for 2H-$MoTe_2$ and 2H-$MoSe_2$ are performed over a wide r-range from $1.5 < r < 50$ Å. Lattice constants and atomic displacement parameters were constrained by hexagonal symmetry. The results of the PDF analysis are summarized in Figure S4. For both temperatures T = 90 K and 300 K, the PDF is in good agreement with the 2H polytype (SG:$P6_3/mmc$) reported by D. Puotinen and R.E. Newnham [9]. No evidence of structural distortions or segregation, originating from the dilute concentration of intrinsic defects was found, in line with the observation of a spatially homogeneous distribution of defects from STM. The best PDF fit for 2H-$MoTe_2$ at 300 K, yields refined lattice parameters

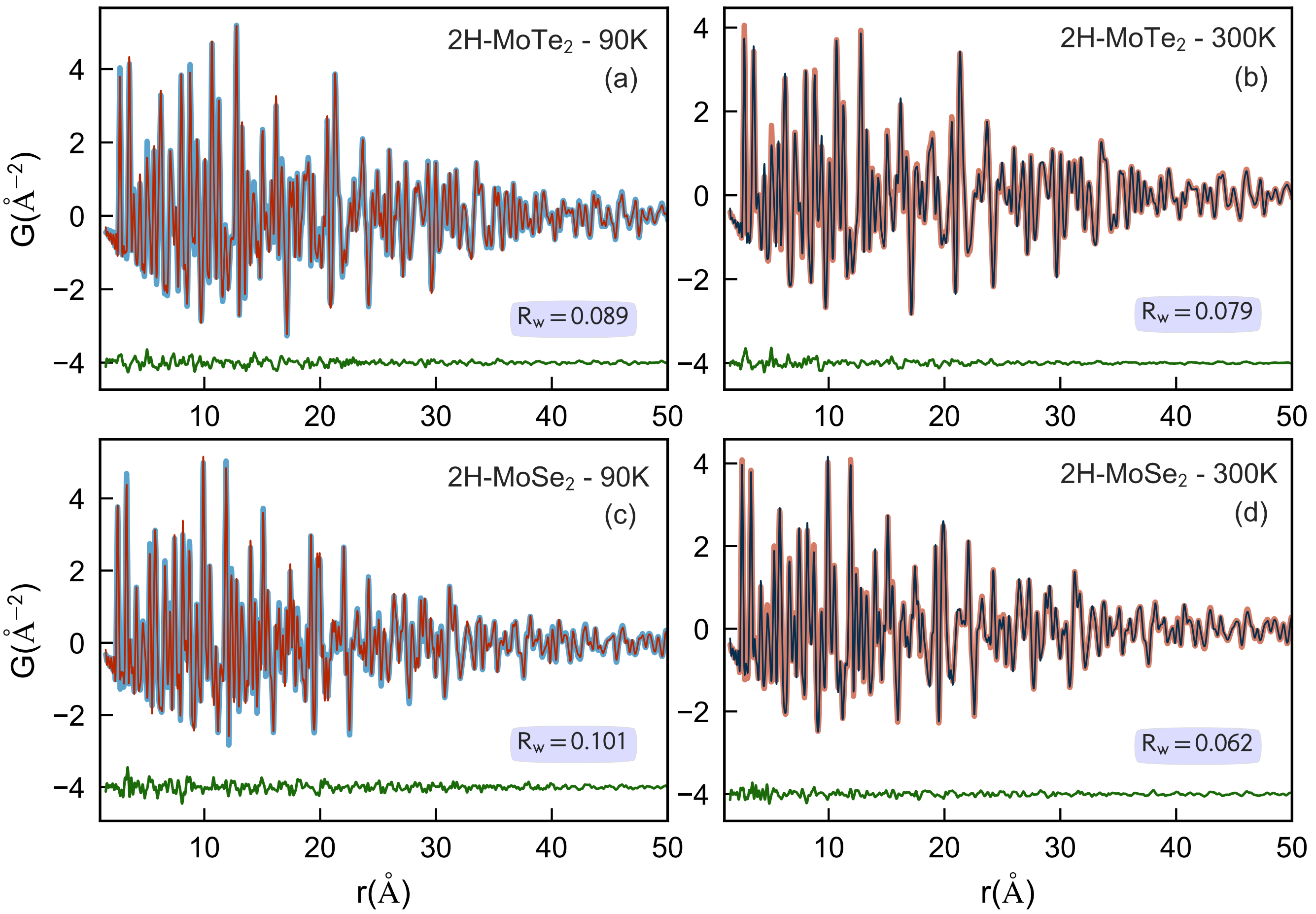

Fig. S4. (Color online) **PDF results for 2H-$MoTe_2$ and 2H-$MoSe_2$.** PDF average structure refinements for 2H-$MoTe_2$ (a,b) and 2H-$MoSe_2$ (c,d) at 90 K and 300 K fit to the hexagonal 2H-structure model. Thick curves are the experimental PDFs, and the refined PDFs are overlayed as thin solid lines. Fit residuals, in green, are offset below.

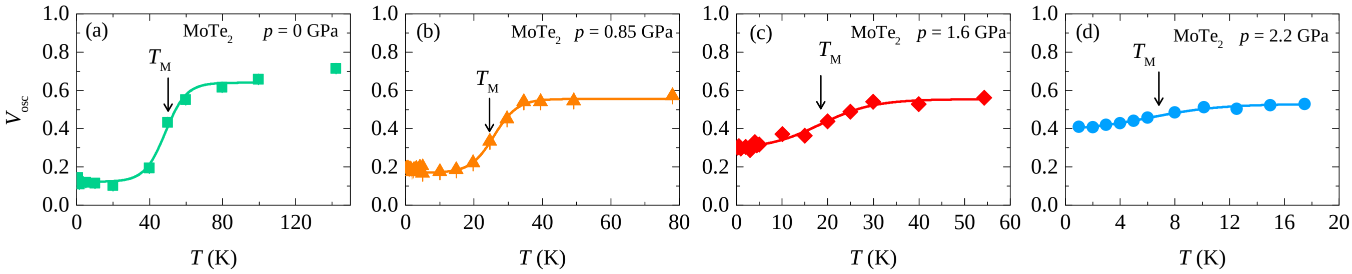

Fig. S5. (Color online) **Temperature and pressure evolution of the paramagnetic fraction** V_{osc}**.** The temperature dependence of the V_{osc} for the polycrystalline sample of $MoTe_2$ at ambient pressure (a) and at various applied pressures (b-d). The solid lines represent fits to the data by means of Eq. (3).

of $a = b = 3.5186$ Å, and $c = 13.9631$ Å. For 2H-$MoSe_2$ at 300 K, $a = b = 3.2875$ Å and $c = 12.9255$ Å.

High pressure μSR data for $MoTe_2$

The results of the high pressure weak-TF μSR experiments are summarised in Figs. S5(a-d). Namely, we plot the temperature dependence of V_{osc} for various hydrostatic pressures. A strong reduction of $T_{\rm M}$ as well as of the corresponding magnetic fraction is observed under pressure. For the highest applied pressure of $p = 1.6$ GPa the T_M decreases by ~ 30 K and the corresponding fraction by ~ 50 %. Note that the pressure dependence of the high temperature magnetic transition was not measured due to technical issues related to the high pressure

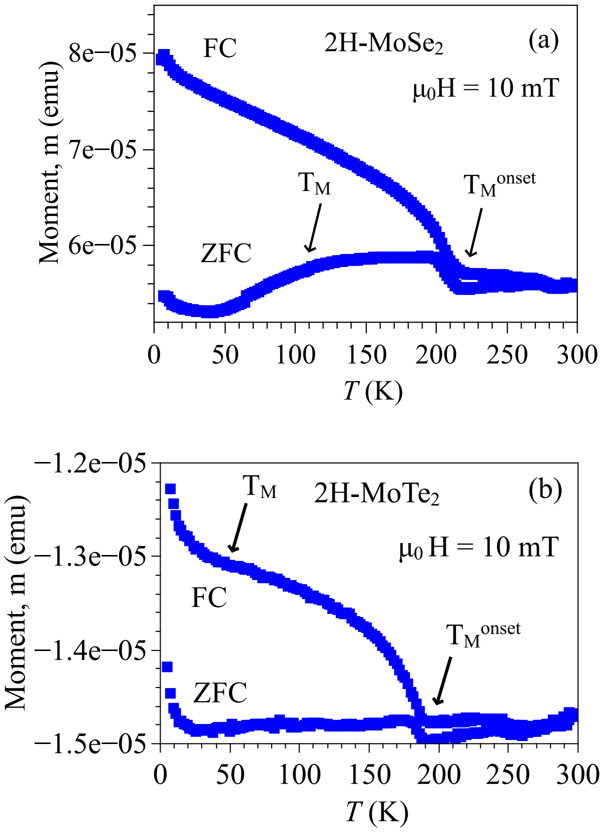

Fig. S6. (Color online) **Magnetization data for $MoSe_2$ and $MoTe_2$.** The temperature dependence of zero-field cooled and field-cooled magnetic moments of $MoSe_2$ (a) and $MoTe_2$ (b), recorded in an applied field of $\mu_0 H = 10$ mT. The arrows mark the onset of the difference between ZFC and FC moment as well as the anomalies seen at low temperatures.

μSR technique.

Magnetization measurements of $MoTe_2$ and $MoSe_2$

We carried out magnetization experiments on both $MoTe_2$ and $MoSe_2$ and measured the temperature dependence of the macroscopic magnetic moment under zero-field cooled (ZFC) (sample was cooled down to the base-T in zero magnetic field and the measurements were done upon warming) as well as field-cooled (FC) conditions (the sample was cooled down to the base-T in an applied magnetic field and the measurements were done upon warming). Figures S6a and b show the moment for $MoSe_2$ and $MoTe_2$, respectively, recorded in an applied field of 10 mT. A large difference between ZFC and FC magnetic moments are seen for both samples. The samples show a combination of a temperature-independent diamagnetism, small van Vleck-type paramagnetism (which is determined by the energy separation of bonding and anti-bonding states E_a - E_b. E_a - E_b is proportional to the band gap E_g) and a ferromagnetic contribution that onsets near 230 K and 180 K for $MoSe_2$ and $MoTe_2$, respectively. The difference between the FC and ZFC response suggests that different ferromagnetic domains tend to cancel out (anti-align) after ZFC. If we FC, then the domains align. The onset temperatures of hysteresis 230 K and 180 K for $MoSe_2$ and $MoTe_2$, respectively, are close to the temperature T_M^{onset} below which μSR experiments show the appearance of inhomogeneous magnetism (see Figure S2). This means that below T_M^{onset} some small ferromagnetic domains (droplets of magnetic order) form, which produces inhomogeneous magnetic fields and would result the absence of coherent oscillations in the μSR signal. Instead, the various magnetic fields produced in the sample will give rise to a strong damping of the muon asymmetry, which we clearly observe at $T_M^{onset} = 180$ K in $MoTe_2$ and $T_M^{onset} = 250$ K in $MoSe_2$. μSR observes homogeneous magnetism below $T_M \simeq 40$ K and 100 K for $MoTe_2$ and $MoSe_2$, respectively, and the anomalies (such as an additional increase of the moment and of the difference) at around these temperatures can also be seen in magnetization data (Figures S6a and b). Thus, the μSR data for oscillations and damping and the SQUID data for temperature and field dependence of the magnetization are completely consistent with each other.

For the sample $MoSe_2$ the magnetic contribution dominates over the diamagnetism, gives rise to total positive moment. In contrary, for $MoTe_2$ diamagnetism dominates over the magnetic contribution. Assuming that the core diamagnetism is nearly the same in these materials, this difference might be related to the stronger magnetism (bigger moments) in $MoSe_2$ than in $MoTe_2$. This is consistent with μSR experiments, showing factor of two higher magnetic ordering temperature and factor of 1.5 higher internal magnetic field in $MoSe_2$ as compared to those in $MoTe_2$.

The field dependence of the magnetic moment for $MoSe_2$ and $MoTe_2$, recorded at three different temperatures are shown in Fig. S7a and b, respectively. Large hysteresis loop is observed for both samples at the base temperature, confirming the presence of ferromagnetism in these semiconductors. The magnitude of the loop decreases with increasing temperature and fully closes at high temperatures. This is consistent with the temperature dependent magnetisation data which shows no difference between FC and ZFC response at temperatures above 230 K and 180 K for $MoSe_2$ and $MoTe_2$, respectively. The coercive field, estimated at $T = 5$ K, is 300 G and 400 G for $MoSe_2$ and $MoTe_2$, respectively.

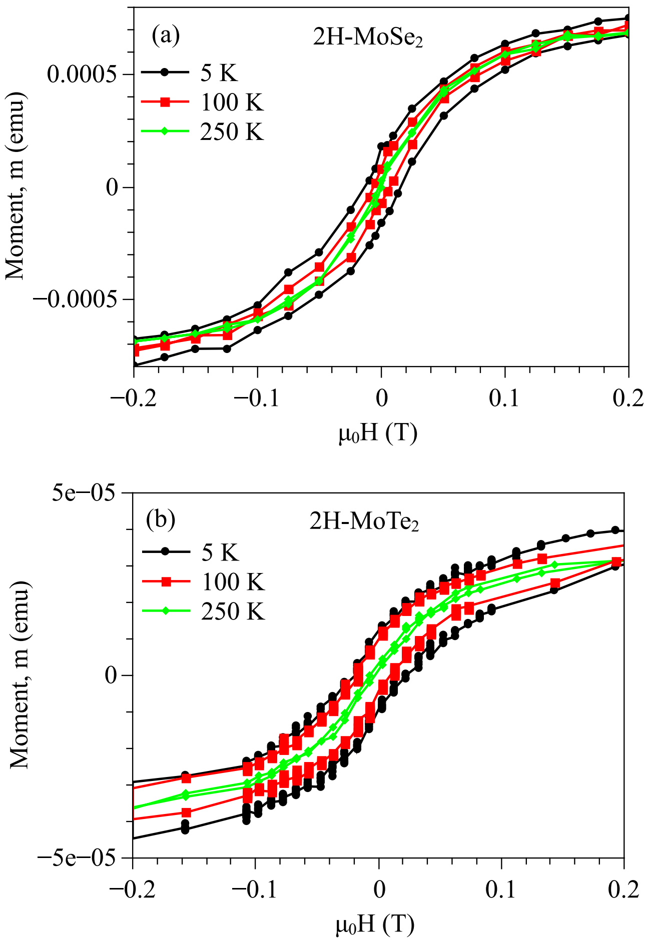

Fig. S7. (Color online) **Hysteresis loop for $MoSe_2$ and $MoTe_2$.** The field dependence of magnetic moment of $MoSe_2$ (a) and $MoTe_2$ (b), recorded at various temperatures.

Local Magnetization of the antisite defect versus Hubbard U

The Hubbard U value used in our simulations is in the range of 0.5 to 4.0 eV to account for strong on-site interactions at the defect as mentioned explicitly in the Methods. We calculated the local magnetization of the antisite defect as a function of U. The strong U dependence of moment is found as shown in Figure S8b. We have also explicitly calculated the magnitude of the Hubbard U using linear response theory as included in Figure S8a. The magnitude obtained is of U_{LR} = 2.72 eV for the antisite defect (this value is marked as the dashed line in Figure S8b), which is within the limit of initial range calculated the magnetic properties of the defects.

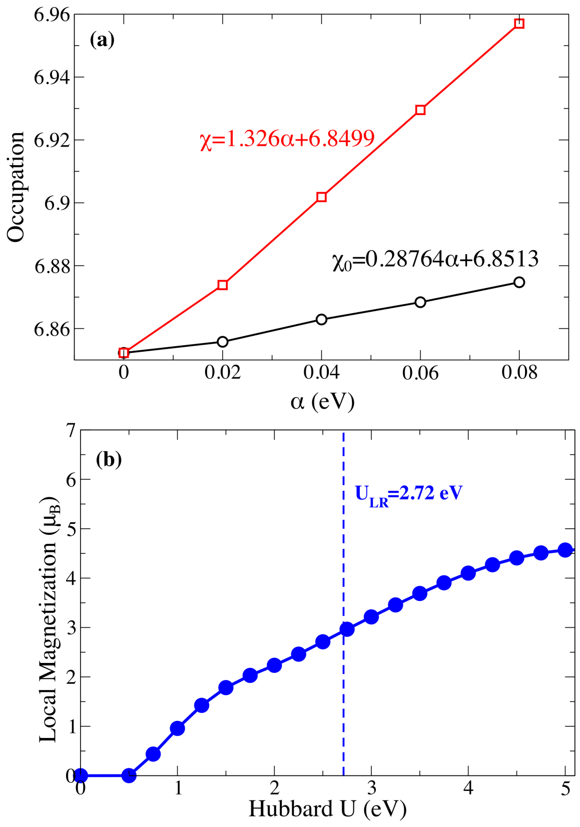

Fig. S8. (Color online) **Calculated magnetization of the antisite defect versus Hubbard U.**(a) Occupation number versus rigid potential shifts α for antisite defects for the bare, non-interacting potential χ_0 and the interacting potential χ. From the angular coefficients of both curves we can extract the optimum U_{LR} for our system, $U_{LR} = {\chi_0}^{-1}$ - χ^{-1} [53]. (b) Variation of the local magnetization at the defect antisite versus U. At $U=$ 0, no magnetic moments are observed as the defect shows a symmetric configuration at the Mo-Mo bonds. At $U >$ 0.5 eV, this symmetry is broken and the defect develops an appreciable magnetic moment that increases with U as a result of the increased localization of the bands.
